# Curcumin Attenuated Neurotoxicity in Sporadic Animal Model of Alzheimer’s Disease

**DOI:** 10.3390/molecules26103011

**Published:** 2021-05-18

**Authors:** Ines ELBini-Dhouib, Raoudha Doghri, Amenallah Ellefi, Imen Degrach, Najet Srairi-Abid, Asma Gati

**Affiliations:** 1 Laboratory of Biomolecules, Venoms and Theranostic Applications, LR20IPT01, Institut Pasteur of Tunis, 1002 Tunis, Tunisia; AmenallahEllafi@pasteur.tn (A.E.); najetsrairi@yahoo.fr (N.S.-A.); 2 Laboratory of Anatomo-Pathology, Institut Salah Azaiez, 1006 Tunis, Tunisia; raoudha.doghri@gmail.com; 3 Animal Unit, Institut Pasteur de Tunis, 1002 Tunis, Tunisia; Imendegrach@pasteur.tn; 4 Laboratory of Genetics, Immunology and Human Pathology, Faculté des Sciences de Tunis, Université Tunis El Manar, 2092 Tunis, Tunisia; asmagati@yahoo.fr

**Keywords:** aluminum, antioxidant, anti-inflammatory, neuroprotective

## Abstract

Alzheimer’s disease (AD) is one of the most common neurodegenerative diseases leading to dementia. Despite research efforts, currently there are no effective pharmacotherapeutic options for the prevention and treatment of AD. Recently, numerous studies highlighted the beneficial effects of curcumin (CUR), a natural polyphenol, in the neuroprotection. Especially, its dual antioxidant and anti-inflammatory properties attracted the interest of researchers. In fact, besides its antioxidant and anti-inflammatory properties, this biomolecule is not degraded in the intestinal tract. Additionally, CUR is able to cross the blood–brain barrier and could therefore to be used to treat neurodegenerative pathologies associated with oxidative stress, inflammation and apoptosis. The present study aimed to assess the ability of CUR to induce neuronal protective and/or recovery effects on a rat model of neurotoxicity induced by aluminum chloride (AlCl_3_), which mimics the sporadic form of Alzheimer’s disease. Our results showed that treatment with CUR enhances pro-oxidant levels, antioxidant enzymes activities and anti-inflammatory cytokine production and decreases apoptotic cells in AlCl_3_-exposed hippocampus rats. Additionally, histopathological analysis of hippocampus revealed the potential of CUR in decreasing the hallmarks in the AlCl_3_-induced AD. We also showed that CUR post-treatment significantly improved the behavioral, oxidative stress and inflammation in AlCl_3_-exposed rats. Taken together, our data presented CUR as a nutraceutical potential through its protective effects that are more interesting than recovery ones in sporadic model of AD.

## 1. Introduction

Alzheimer’s disease (AD), a chronic neurodegenerative disease, is one of the most prevalent types of dementia. AD is characterized by selective neuronal loss, and cognitive impairment caused by the accumulation of amyloid β (Aβ) protein plaques [1]. The etiology of AD has not been totally discovered yet; however, both genetic and environmental factors have been shown to play a vital role [2]. Numerous lines of evidence showed that aluminum (Al), as an environmental toxin, could act as a causative factor for AD development [3,4]. In fact, aluminum possesses a neuro/cholinotoxin-like effect that affect neuronal structure [5,6], blood–brain barrier (BBB) permeability and the cholinergic/noradrenergic neurotransmission [7,8,9].

Several studies showed that the exposure to solid form (such as aluminum chloride) and its decomposed form (ion metal Al^3+^) could: (i) alter BBB, (ii) affect the axonal transports and (iii) induce inflammatory responses as well as synaptic structural abnormalities, resulting in profound memory loss [4,7,8,9]. Additionally, the metal ions Al^3+^ accelerate the dynamic process of Aβ aggregation, thus enhancing the neurotoxicity on the neuronal cells, as a consequence of marked alterations of biophysical properties of Aβ peptide which leads to its accumulation in the cortex and the hippocampus [4,9,10].

The potential neurotoxicity effect of Al was also confirmed on experimental animal models [3,11], showing that chronic exposure to chloride form of aluminum (AlCl_3_) causes neurologic signs which mimic progressive neurodegeneration in the hippocampus, cerebral cortex, and spinal cord [9,12]. Thus, animal treated with AlCl_3_ continuously, might be a reliable model for studying the sporadic form of AD and the mechanisms underlying this pathology and may be used to screen the impact of new drugs in the development of AD [11].

Actually, treatment strategies against AD are limited and many clinical trials have been conducted to clear misfolded β-amyloid proteins from brain tissues, but without additional outcomes. For instance, acetylcholinesterase (AChE) inhibitors, including donepezil or noncompetitive NMDA receptor antagonists, are approved to treat this pathology [13]. However, these drugs were not able to slow down disease progression and caused adverse effects, such as diarrhea, nausea or insomnia [14]. Therefore, the development of more effective medications for AD treatment is presently under extensive investigations.

Molecules derived from plant extracts are currently considered as potential complementary and/or alternative therapies against AD. Indeed, the use of antioxidant agents from diet showed a significant therapeutic effect on various neurodegenerative disorders associated with oxidative stress [15]. Curcumin (CUR), a major compound of curcuma, was extensively studied for its antioxidant, anticancer, anti-inflammatory and cytoprotective properties [15,16,17]. Particularly, CUR was shown to provide neuroprotection by inducing the upregulation of the transcription of nuclear factor (erythroid-derived 2)-like 2 (Nrf2) and by suppression of NF-κB activation in cellular and animal models of neurodegenerative disorders [18,19]. Moreover, CUR enhanced neurogenesis in chronically stressed rats [20], in aged rats [21] and in Aβ-induced model of AD [22]. The relation between the bioaccumulation of Al in the brain and neurotoxicity was not fully understood and the effects of prolonged CUR treatment on neurogenesis were not yet studied in the sporadic AD. Therefore, the main objective of the present study was to investigate the potential of CUR on the protection/recovery effects against AlCl_3_-induced AD.

## 2. Results

### 2.1. CUR Treatment Acts on Body and Organ Weights Affected by AlCl_3_ Administration

We first checked if AlCl_3_ and CUR, at used doses, induce toxicity on phase I and II treatment. We found that, besides no mortality in any rat groups was recorded, no significant difference in body weight was noticed (data not shown). We also did not notice any significant difference in final weights between CTR− and CTR+ rat groups (*p* < 0.05) (Table 1). However, as shown in Table 1, an increase on the final weight of CUR1 group compared to CTR+ one; and a significant increase in final body weight of CUR2 group in comparison to control group CTRAl was observed.

On the other hand, weight gain increased on CTR+ rats, and was significantly lower in the CUR groups when compared with those of CTR+ and CTRAl groups (*p* < 0.05) (Table 1). However, food efficiency ratio (FER) in CUR1 and CUR2 groups remains invariable when compared with the control groups (CTR+ and CTRAl) (*p* < 0.05).

Besides, a decrease of absolute hippocampus weight on CTR+ rat group was noticed; whereas no significant difference was observed in the other groups (Table 1).

### 2.2. CUR Protects against AlCl_3_-Induced Cognitive Impairment

**Figure 1:**
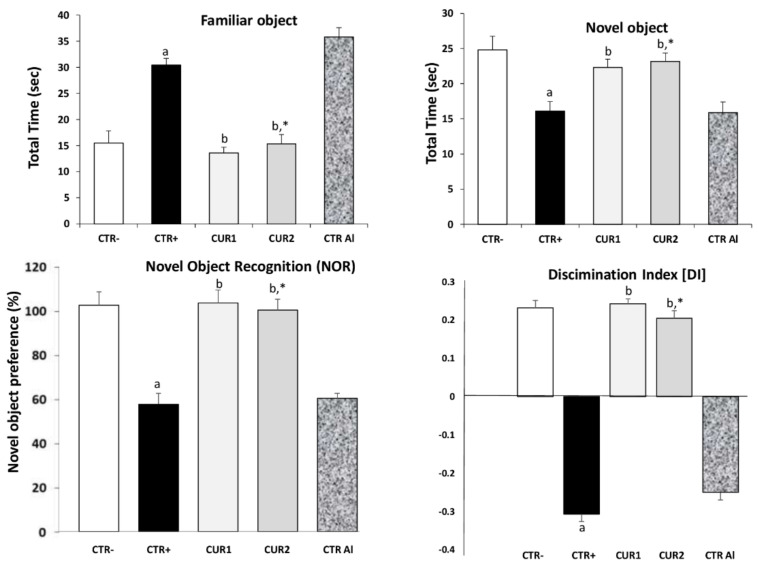
Effect of co and post-treatment of curcumin on cognitive impairment on rat model of Alzheimer’s Disease (AD) induced by chronic administration of AlCl_3_. Values are mean ± SEM. Note: CTR−: negative control group; CTR+: positive control group (treated by aluminum chloride (AlCl_3_)); CUR1: co-treatment with curcumin; CUR2: post-treatment with curcumin; CTRAl: exposed to AlCl_3_ for 90 days and survive 60 days without any treatment; a: *p* < 0.05 as compared to CTR− group; b: *p* < 0.05 as compared to CTR+ group; *: *p* < 0.05 as compared to CTRAl group, repeated measures two-way ANOVA followed by Tukey’s test for multiple comparisons.

Assessment of memory of rats exposed to AlCl_3_ and CUR by novel object recognition (NOR) test is presented in Figure 1. A marked impairment in memory recognition was recorded in AlCl_3_ exposed rats (*p* < 0.01), in comparison with CTR− rat group. As shown in Figure 1, AlCl_3_ exposed rats spent more time on sniffing familiar object rather than novel object. This alteration of memory recognition persisted even after stopping treatment. Interestingly, co and post-treatments with CUR significantly improved memory recognition and rescue memory deficit (*p* < 0.001), showing that CUR protects against AlCl_3_-induced non-spatial memory deficit in the NOR task.

### 2.3. CUR Protects against AlCl_3_-Induced Anxiety

**Figure 2:**
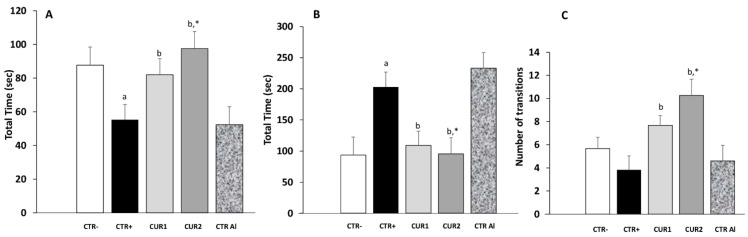
Effect of co and post-treatment of curcumin on the anxiety in the rat model of AD induced by chronic administration of AlCl_3_. (**A**) time spent in the white area; (**B**) Time spent in the black area; (**C**) number of transitions between white and black areas. Values are mean ± SEM. Note: CTR−: negative control group; CTR+: positive control group (treated by aluminum chloride (AlCl_3_)); CUR1: co-treatment with curcumin; CUR2: post-treatment with curcumin; CTRAl: exposed to AlCl_3_ for 90 days and survive 60 days without any treatment; a: *p* < 0.05 as compared to CTR− group; b: *p* < 0.05 as compared to CTR+ group; *: *p* < 0.05 as compared to CTRAl group, repeated measures two-way ANOVA followed by Tukey’s test for multiple comparisons.

In this task, an anxiety-like profile is characterized by increased time spent in the dark compartment and decreased transitions between light and dark compartments. This profile was showed on CTR+ rats. In our data, CUR1 group spent 52.33% of time in the dark compartment (Figure 2). This value increased by 37% in rats of the CTR+ group. In phase II of the experiment, the time spent in dark compartment increased slightly in CTRAl rats when compared with CTR+ suggesting that prolonged exposure to AlCl_3_ favors anxiety. The CUR treatment alleviates the time spent in dark compartment in CUR1 as well as in CUR2 groups.

Anxiety level was also assessed by counting the number of box switches. As shown in Figure 2, the mean number of transition during the light/dark box test was significantly higher in CUR1 and CUR2 groups in comparison to all control groups. Taken together, these results suggested that CUR protects against anxiety.

### 2.4. CUR Improves AlCl_3_-Induced AChE Alteration

**Figure 3:**
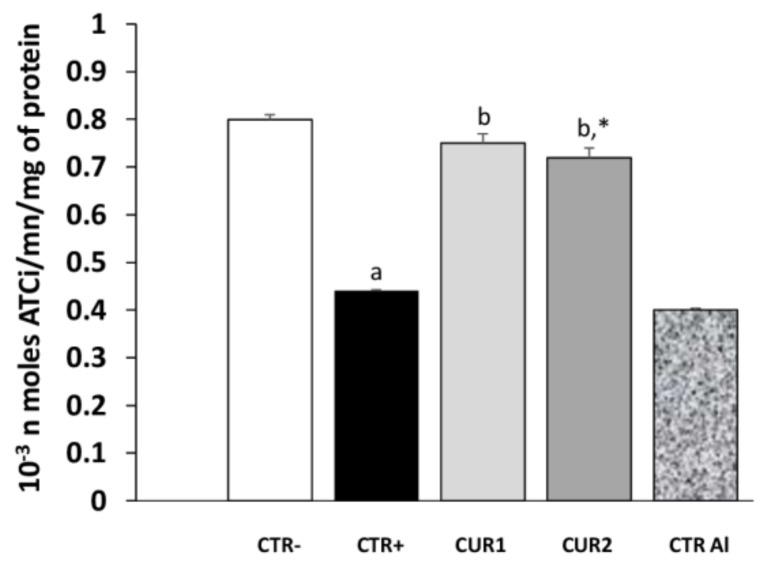
Effect of co and post-treatment of curcumin on the hippocampus acetylcholinesterase activity on rat model of AD induced by chronic administration of AlCl_3_.Values are mean ± SEM. Note: CTR−: negative control group; CTR+: positive control group (treated by aluminum chloride (AlCl_3_)); CUR1: co-treatment with curcumin; CUR2: post-treatment with curcumin; CTRAl: exposed to AlCl_3_ for 90 days and survive 60 days without any treatment; a: *p* < 0.05 as compared to CTR- group; b: *p* < 0.05 as compared to CTR+ group; *: *p* < 0.05 as compared to CTRAl group, repeated measures two-way ANOVA followed by Tukey’s test for multiple comparisons.

We evaluated the effect of long-term exposure to AlCl_3_ on acetylcholinesterase (AChE) activity. As shown in Figure 3, aluminum chloride treatment significantly decreased AChE activity in hippocampus, as compared to control rats; and its impact continues after the intake of AlCl_3_. Interestingly, co and post-treatments with CUR restored AChE activity significantly.

### 2.5. CUR Nullifies AlCl_3_-Induced Oxidative Stress

Oxidant and antioxidant parameters in the hippocampus are reported in Table 2. We noticed that the levels of MDA were significantly elevated and the activities of SOD and catalase were significantly decreased in the hippocampus in CTR+ rats compared to CTR− animals. Co-treatment with CUR significantly attenuated oxidative stress in the hippocampus by decreasing levels of MDA and enhancing SOD and catalase activities, when compared to AlCl_3_-treated animals.

As shown in Table 2, after stopping AlCl_3_ treatment, MDA level in CTRAl group continued to increase (*p* < 0.01). Whereas, the MDA level in CUR2 group decreased compared to CTRAl group (*p* < 0.01). Additionally, SOD and catalase activities continued to decrease in CTRAl group (*p* < 0.01). In CUR2 group, SOD and catalase activities increased when compared to those of CTRAl group (*p* < 0.05). These results demonstrated that co and post-treatments of CUR attenuate AlCl_3_-induced oxidative stress in the animal hippocampus.

### 2.6. CUR Protects against AlCl_3_-Induced Neuroinflammation

**Figure 4:**
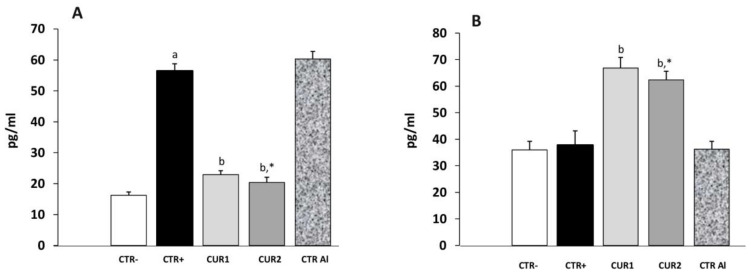
Effect of co and post-treatment of curcumin on the pro and anti-inflammatory cytokines levels on the hippocampus of AD-rat model induced by chronic administration of AlCl_3_: (**A**) effect on the INF-γ concentration; (**B**) effect on the IL-4 concentration. Values are mean ± SEM. Note: CTR−: negative control group; CTR+: positive control group (treated by aluminum chloride (AlCl_3_)); CUR1: co-treatment with curcumin; CUR2: post-treatment with curcumin; CTRAl: exposed to AlCl_3_ for 90 days and survive 60 days without any treatment; a: *p* < 0.05 as compared to CTR− group; b: *p <* 0.05 as compared to CTR+ group; *: *p* < 0.05 as compared to CTRAl group, repeated measures two-way ANOVA followed by Tukey’s test for multiple comparisons.

As the INF-γ contributes to neuropathogenic processes of AD, we were interested on CUR treatment impact on this cytokine level. As shown in Figure 4, INF-γ concentration was higher in the rat hippocampus of CTR+ when compared with that of negative control group CTR− (*p* < 0.01). Interestingly, co- and post-treatments with CUR significantly reduced INF-γ concentration, suggesting that CUR administration attenuates AlCl_3_-induced inflammation in the hippocampus. Besides, the IL-4 concentration in the hippocampus was studied in different rat groups. As shown in Figure 4, IL-4 concentration did not differ in CTR+, as well as in CTRAl groups, in comparison with CTR− one. However, our results showed that co- and post-treatments of CUR reduce IL-4 concentration. Hence, these results suggest that CUR inhibits the production of pro-inflammatory cytokine and stimulates that of anti-inflammatory cytokine in the hippocampus.

### 2.7. CUR Enhances Viability of Hippocampus Cells in AlCl_3_-Exposed Rats

Severals studies showed that apoptotic mechanisms might be involved in AD pathogenesis. In this context, we investigated the effect of CUR effect on the viability of hippocampus cells of AlCl_3_-exposed rats.

**Figure 5:**
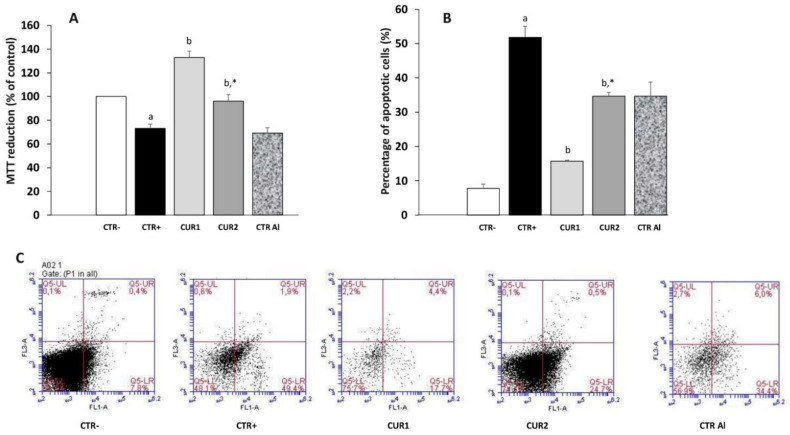
Effect of co and post-treatment of curcumin on the hippocampus cell viability and apoptosis on rat model of AD induced by chronic administration of AlCl_3_: (**A**) effect on the percentage of cell viability by MTT test; (**B**) effect on the percentage of apoptosis by annexinV-FITC/PI staining; (**C**) apoptosis was detected by cytometry using the Annexin V-FITC/PI Apoptosis Detection kit (viable cells are negative to both probes; apoptotic cells are Annexin positive). Values are mean ± SEM. Note: CTR−: negative control group; CTR+: positive control group (treated by aluminum chloride (AlCl_3_)); CUR1: co-treatment with curcumin; CUR2: post-treatment with curcumin; CTRAl: exposed to AlCl_3_ for 90 days and survive 60 days without any treatment; a: *p* < 0.05 as compared to CTR− group; b: *p* < 0.05 as compared to CTR+ group; *: *p* < 0.05 as compared to CTRAl group, repeated measures two-way ANOVA followed by Tukey’s test for multiple comparisons.

First, neuronal viability following AlCl_3_ exposure was determined by MTT test (Figure 5A). As reported in Figure 5A, AlCl_3_ induces cytotoxicity of hippocampus cells (*p* < 0.01) whereas CUR treatment restores cell viability in CUR1 group. Interestingly, the neuronal viability alteration is maintained after stopping AlCl_3_ exposure (CTRAl group) whereas neuronal viability was enhanced by post-treatment of CUR.

Using Annexin V/PI staining to detect cell death in hippocampus, we confirmed 22.1% and 25.1% inhibition of apoptosis of hippocampus cells with co- and post-treatments with CUR, respectively, versus 40.4% in AlCl_3_-exposed rats (Figure 5B). Taken together, these results suggest that CUR enhances viability of hippocampus cells in AlCl_3_-exposed rats (Figure 5B,C).

### 2.8. CUR Reduced Neurodegenetration in the Hippocampus of AlCl_3_-Exposed Rats

In order to evaluate AlCl_3_ toxicity and therapeutic/recovery effects of CUR, histopathological analysis was performed in the hippocampus of different groups of rats.

**Figure 6:**
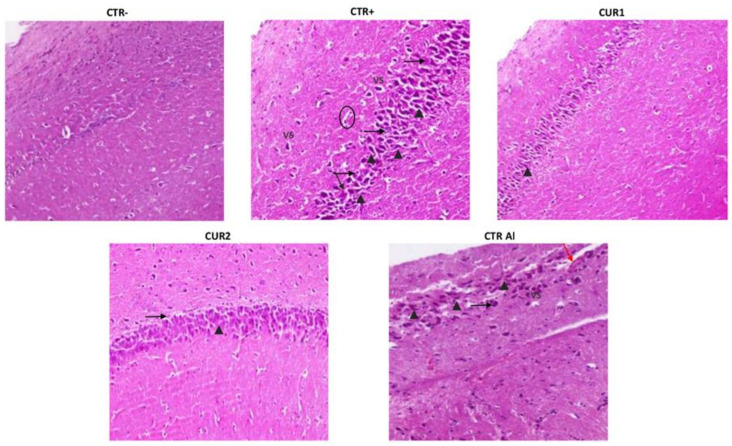
Effect of co and post-treatment of curcumin on histological examination of the hippocampus on rat model of AD induced by chronic administration of AlCl3. The red arrow indicates apoptosis. The black arrowhead indicates necrosis and pyk-notic nuclei. The black arrow indicates lymphocyte infiltration. VS indicates severe congestion in the karyopyknosis of neurons. Note: CTR−: negative control group; CTR+: positive control group (treated by aluminum chloride (AlCl3)); CUR1: co-treatment with curcumin; CUR2: post-treatment with curcumin; CTRAl: exposed to AlCl3 for 90 days and survive 60 days without any treatment.

As shown in Figure 6, AlCl_3_-induced injury on the histological architecture, as evidenced by the presence of severe congestion in the karyopyknosis of neurons (cells with darkened nuclei in the CA1 area of the hippocampus), in comparison to the CTR− group. In addition, AlCl_3_ causes the formation of neurofibrillary tangles in the hippocampus. Other sections showed neuronal degeneration and pyknosis in CA1 area shown by black arrows. Vacuolated cytoplasm as marker of neurodegeneration was observed. Amyloid plaque-like structures (shown by circle) were also observed in hippocampal sections. Hippocampal lesions persisted on CTRAl group (Figure 6).

Interestingly, the CUR treatment restored the normal morphological phenotype of neurons manifested by rounded neuronal build, normal cytoplasm and well-defined nuclei. In addition, a relatively normal morphological appearance of the nerve cells with few enlarged pericellular space and necrosis were noticed in the hippocampal neurons of the CUR1 rats group. Recovery effect of CUR has been only highlighted by reduced karyopyknotic neuronal cells. In addition, the post-treatment with CUR slightly reduced the neurofibrillary tangles formation and neutrophil infiltration. Yet, CA1 pyramidal neurons’ necrosis vacuolizations are observed in CUR2 hippocampus. These results showed a better benefit of CUR co-treatment than post-treatment strategy (Figure 6).

## 3. Discussion

Previous reports have suggested a potential relationship between the aluminum-induced neurotoxicity and the physiopathology of AD [4,7,8]. This can be related to the increase in the use of aluminum containing drugs (such as antacids and vaccine adjuvants) and food additives; resulting to increase the amount of Al exposed to the body. For that, several scientific teams actually used the aluminum chloride form as a neurotoxic agent to induce a sporadic model of AD. In addition, this model is increasingly used to study bioactive/chemical molecules potential on the treatment of this pathology and to follow up drug intervention studies. In fact, data on AlCl_3_ exposed mice are consistent with the main pathological features (oxidative stress, inflammation and apoptosis) of clinical AD [23,24], indicating that the sporadic AD rat model used in this work meets the experimental AD study requirements. Thus, in the present study, we focused on the therapeutic and recovery effects of CUR on chronic AlCl_3_-exposed rats. We also investigated the persistence of pathological signs after stopping AlCl_3_ administration. Indeed, the research originality of the present study is (i) to compare, for the first time, the therapeutic and recovery effects of curcumin in sporadic models of AD and (ii) to show the repercussion of aluminum chloride bioaccumulation in the rat hippocampus.

Our results showed that chronic exposure to aluminum chloride induced a significant loss of body and hippocampi weights. This result is due to the general toxicity of AlCl_3_ and its accumulation in the brain [4,8]. Interestingly, co-treatment with 100 mg/kg of CUR significantly restores body and organ weights.

We also demonstrated that CUR treatments protect rats against deterioration of spatial memory and anxiety-related behavior, induced by a long-term exposure to AlCl3.

Our results also noticed memory impairment and neurobehavioral deficits on positive groups (CTR+ and CTRAl) which persist even after stopping AlCl_3_ administration. These disruptions have been also described by Thirunavukkarasu et al. [25].

Behavioral activities have been found to be associated with acethylcholine level in the brain. Indeed, a strong decrease in choline acetyltransferase activity in the cortex and hippocampus has been related to human memory and cognitive dysfunction in AD patients [26]. Our data showed that AChE activity decreased in hippocampus of AlCl_3_-exposed rats, which corroborate with recent studies suggesting that the administration of AlCl_3_ decreases acetylcholinesterase activity in mouse [23] and rat brains [25]. Recently, Martinez et al. showed that the dose of 100 mg/kg of AlCl_3_ inhibits AChE activity on the hippocampus of rat after 60 days of treatment, confirming the inhibition of cholinergic activity [27]. All these studies advocate that the deterioration of cholinergic neurotransmission is mainly related to the accumulation of acetylcholine on the brain of AD patients [5,28]. Interestingly, as we showed in our work that CUR restored the altered AChE activity significantly in the hippocampi of rats and consequently rehabilitated rats behaviors.

On the other hand, it has been reported that AlCl_3_ exposure was correlated with the induction of oxidative stress leading to the accumulation of free radical, which could be responsible of severe DNA damage, lipid peroxidation, neuronal cell apoptosis and might enhance brain cell injury [5,25]. Our results showed that in AlCl_3_ exposed rats, the level of MDA increased, while those of CAT and SOD decreased. Interestingly, CUR reversed these perturbations and decreased the antioxidant capacity even than that of negative control rats. This finding support previous studies demonstrating the antioxidant effects of CUR on various stress and toxic animal models [29,30]. Indeed, CUR, because of its lipophilic property, crosses the BBB and acts in situ to inhibit lipid peroxidation and to enhance endogenous antioxidant mechanisms [30,31,32]. In fact, the structure of curcumin contains diketone and phenol as reactive functional groups that participate to (i) scavenge the reactive oxygen species (ROS) and (ii) mimic antioxidant enzymes [33]. Therefore, CUR administration, via its antioxidation therapy, could be used as an important strategy to treat neurodegeneration processing.

Moreover, we investigated the brain inflammation, since it is described as a pathological hallmark of AD, characterized by an imbalance between pro-inflammatory and anti-inflammatory cytokines. Herein, we demonstrated that co and post-treatments of CUR-suppressed INF-γ production and increased IL-4 secretion in hippocampus of AlCl_3_ exposed rats. These results corroborate with others showing that CUR modulated the inflammatory status by the (i) inhibition of TNF-α and IL-1β production in the rat brain, (ii) suppression of the release of IL-6 and TNF-α and (iii) induction of IL-4 production by microglial cells [34]. Our results also demonstrated that co and post-administration of CUR significantly reduced (i) neuronal injury, (ii) number of karyopyknosis of neuronal cells, (iii) vacuolated cytoplasm and (iv) cellular depletion. It is evident, from the obtained results, that the anti-inflammatory effect of CUR enhances neuronal cell structure in the rat hippocampus. Further, different mechanisms might be involved during CUR treatment such as the activation of activating protein-1 and nuclear factor kappa B in monocytes or macrophages [35], the induction of cyclo-oxygenase-2 [36], the activation of the antioxidant protein Heme Oxygenase 1 (HO-1) [37] and the reduction of mitochondrial dysfunction [38,39].

Neuronal apoptosis has been described in several CNS disorders and is involved in brain neurodegeneration. Therefore, we addressed the effect of CUR on hippocampus cells of AlCl_3_-treated rat and showed that CUR enhances viability of hippocampus cells in AlCl_3_-exposed rat. Our data confirms results showing that CUR was able to protect cortical neurons from tert-butyl hydroperoxide (t-BHP)-induced apoptosis in rat cortical neurons [38] and attenuated cell apoptosis induced in SH-SY5Y cells (in vitro model of neuroscience research, particularly the Alzheimer’s disease field) [40,41]. So far, apoptosis has been targeted to develop neuroprotective drugs. Delightfully, CUR prevents the neuronal cell apoptosis, maintains neurons number and reduces the progression of AD.

Summing up, curcumin has the potential to be a modulator of neuro-inflammation, apoptosis and oxidative stress pathways that are involved in AD model induced by chronic exposure to AlCl_3_. Hence, CUR forms several types of complexes with Al^3+^ depending on the stoichiometry of the reaction condition [42]. In fact, the interaction between Al^3+^ and curcumin is capable to scavenge Al^3+^, which is toxic for neuron, and prevents the interaction with Aβ protein [33,43], therefore, reducing the ability to induce AD, as demonstrated in our study.

Interestingly, we showed for the first time that the functional disturbance induced by AlCl_3_ in the rat hippocampus persists after stopping treatment, confirming the bioaccumulation of AlCl_3_ in the brain. In fact, it was reported that AD patients overloaded with aluminum showed high concentrations of this metal in the central nervous system [44], although aluminum is reported to accumulate in basal forebrain and cerebellum. Moreover, our study showed that the hippocampus is also vulnerable for aluminum toxicity.

## 4. Material and Methods

### 4.1. Animal Care and Chemicals

Male adult Wistar albino rats (145 ± 3 g) were housed in clean polypropylene cages and maintained at room temperature (23−25 °C) with humidity range of 40%–70%, with alternating 12-h light and dark cycles. The animals were fed with a standard pellet diet and clean drinking water. All procedures were carried out in accordance with the guidelines for care and use of laboratory animals. After, one-week of acclimatization, animals were randomly divided into groups based on their body weight.

The solutions of aluminum chloride (AlCl_3_) and curcumin (CUR), from Sigma chemicals, were freshly prepared at the beginning of each experiment. Administration of chemicals was given daily at 10:00 a.m., while behavioral tests were performed at 07:00 p.m.

### 4.2. Induction of Neurotoxicity and Treatment Schedule

**Figure 7:**
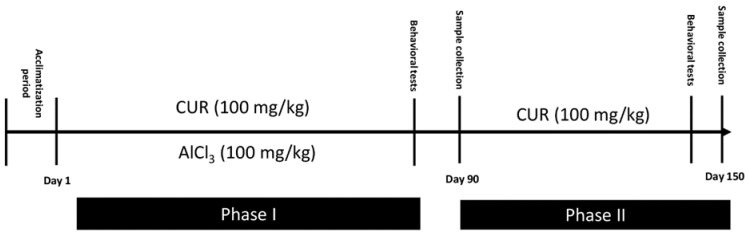
Illustration of the protocol: Timeline showing a summary of the experimental design.

The experiment was conducted in two phases (Figure 7). Phase I was carried out to evaluate the prevention effect of curcumin. Rats were divided into three groups:

- Group CTR−: Six rats served as the control and provided only the vehicle solution (PBS; 1X) for 90 days.
- Group CTR+: Eighteen rats received AlCl_3_ (100 mg/kg b.w) diluted in 1 mL of PBS by oral gavage for 90 days to establish a model of neurotoxicity. At the end of AlCl_3_ treatment and AD induction, 6 of these rats were randomly sacrificed. These rats served as the positive control CTR+ of phase I. The other 12 rats continue the phase II of the experiment.
- Group CUR1: In this group, 6 rats were exposed daily to AlCl_3_ (100 mg/kg b.w) diluted in 1 mL of PBS by oral gavage, then treated, after 1 h, with CUR (100 mg/kg b.w) dissolved in 1 mL of corn oil by intragastric administration, for 90 days.

Phase II was carried out for a total period of 150 days (22 weeks), to evaluate the post-treatment effect of CUR rats, which received AlCl_3_ (100 mg/kg b.w) for 90 days. Twelve rats were divided into two groups (*n* = 6):

- Group CTR Al: Six rats were maintained without any other treatment for 60 days and served as a control for the CUR2 group.
- Group CUR2: Six rats were treated with 100 mg/kg of CUR dissolved in 1 mL of corn oil by intragastric administration for 60 days.

The doses of CUR and AlCl_3_ were selected based on those reported in literature [11,15].

The dose of 100 mg/kg of AlCl_3_ was equivalent to 0.74 mM/kg, whereas that of curcumin is equivalent to 0.27mM/kg. Therefore, aluminum ion could bind three molecules of curcumin. In fact, several studies have used the dose of 100 mg/kg/b.w. of AlCl_3_ that was considered sufficient to accelerate the process of neurodegeneration on experimental animal model [45,46,47,48].

### 4.3. Evaluation of Neurodegenerescence

Animals were weighed at the beginning of the experiment, as well as 3 times per week and before sacrifice. Behavioral observations were recorded before and at the end of the experiment. All the behavioral tests were performed in soundless observation and a dimly illuminated room.

#### 4.3.1. Body Weight Change

During the experimental period, the body weight was measured every 2 days, and the dietary weight was measured every 3 days at a constant time frame. Weight gain was calculated by subtracting the initial weight from the final body weight and dividing it by the total breeding period. Food efficiency ratio (FER) was calculated by dividing the total weight gain by the total food intake for 90 days.

#### 4.3.2. Behavioral Assessment

Novel object recognition (NOR) task: NOR was used to assess recognition memory in rats in terms of discrimination index by monitoring sniffing time for familiar and novel objects. The assay was carried out as described by Giorgetty et al., with some modifications [49]. This consisted of three phases: habituation, training and test phases. One day before the trials, the rats were familiarized for 5 min in the device prior to the first NOR trial. During the acquisition trial, 5 min were given to rats to explore two similar cylindrical (A1 and A2) as familiar objects. During the retention trial, one familiar object (A2) was substituted by a novel object termed object B (square), and the rats were permitted to explore both objects for five supplementary minutes. The time taken by rats to explore the two objects during the acquisition and retention phases of the test were recorded manually and separately with two stop watches. Object recognition was computed by using the following formula: Time spent to explore the novel object divided by total time spent to explore both objects (familiar and novel) ×100. Data were analyzed by a one-way ANOVA. Discrimination Index (DI) was then calculated for the retention trial as Equation (1):

DI = B − A1/B + A1 (B = novel object, A1 = familiar object)(1)

Anxiety by light/dark box task: Anxiety of animals from five groups was assessed by light/dark box (LDB), as described by Fernández et al., (2017) [50] with little modifications. Animals were first placed in the light compartment, facing away from the dark compartment. The test was videotaped for 5 min, and subsequently analyzed by a trained observer who recorded the following parameters: latency (in seconds) to enter the dark compartment, time (in seconds) spent in the white compartment and number of transitions between light and dark compartments.

### 4.4. Hippocampus Preparation

Upon completion of behavior tests, rats were sacrificed by decapitation and the brains were immediately removed and washed thoroughly with ice-cold saline and kept at −80 °C until use. After freezing, the hippocampus was removed and rapidly homogenized in 50mM Tris–Cl, pH 7.4 (1:5, *w/v*). The homogenate was centrifuged at 24,000× *g* for 15 min at 4 °C and a fresh low-speed supernatant fraction was used for the measurement of INF-γ and IL-4 concentrations [51]. For biochemical parameters, hippocampus tissues were homogenized in PBS (10×) [15]. For cell death evaluation, cells were isolated, mechanically and aseptically, from the hippocampus of control and treated rats and cultured in DMEM medium (Gibco Life Technologies, Cergy-Pontoise, France) with 10% FBS (Gibco, Gaithersburg, MD, USA). For histological study, the hippocampus of rats from different groups were removed quickly and postfixed in paraformaldehyde solution.

#### 4.4.1. Measurement of Hippocampus INF-γ and IL-4 Concentrations

The contents of INF-γ and IL-4 concentrations in hippocampus were measured using an ELISA kit (Neogen, Lexington, KY, USA) according to the manufacturer’s instruction manuals. The absorbance was measured at 450 nm, and the result was expressed as pg/mg of protein.

#### 4.4.2. Neurochemical Evaluation

Tissue acetylcholinesterase activity was determined at 25 °C in phosphate buffer Tris (0.1 M; pH 7.4) with 0.3 mM DTNB using 1.0 mM ATCh by the Ellman spectrophotometric method [52].

#### 4.4.3. Oxidative Stress Determination

Lipid peroxidation is detected by the determination of malondialdehyde (MDA) production in tissue with the method published by Begue and Aust [53]. Tissue superoxide dismutase (SOD) activity was assayed according to Misra and Fridovich [54]. Tissue catalase (CAT) activity was assayed as described by Aebi (1984) [55].

#### 4.4.4. MTT Cell Viability Assessment

MTT (3[4,5-dimethylthiazol-2-yl]-2,5-diphenyltetrazolium bromide) assay was used to evaluate cellular viability. The MTT solution was added to the cells isolated from the hippocampus (10^3^ cells/well) and incubated for 4 h at 5% CO_2_, 37 °C. Afterward, dimethyl sulfoxide (DMSO, 100 μL, 10 min) was applied to terminate the reaction. The formazan formation was spectrophotometrically analyzed at 570 nm. Results are indicated as percentage of reduction viability taken as reference control rats for which 100% viability was attributed.

#### 4.4.5. Flow Cytometric Detection of Apoptotic Cells in the Hippocampus

Annexin V/Propidium Iodide (PI) staining was performed according to the manufacturer’s instructions for the Annexin V-FITC Apoptosis Detection Kit (BD Pharmingen, Heidelberg, Germany). In brief, rat hippocampus tissue was sectioned and rehydrated. In total, 2 × 10^5^ cells were collected by binding buffer, dyed at 25 °C and were plated on 6-well dishes.

A staining solution containing Annexin V-FITC conjugate solution (195 μL), Annexin V-FITC reaction solution (5 μL) and propidium iodide (10 μL) was prepared in a centrifuge tube and gently mixed. The cells were subsequently incubated with the solution in the dark at room temperature for 20 min. Analysis was performed with Accuri C6 Flow cytometer (BD).

Apoptotic cells were represented by cell that were Annexin V positive and PI negative. Viable cells were represented by cells that were Annexin V negative and PI negative.

#### 4.4.6. Histological Examination

Small pieces of the all rat hippocampus were fixed in 10% of paraformaldehyde solution. The samples were dehydrated with increasing ethanol concentrations, washed in xylene and embedded in paraffin. Paraffin blocks were cut 4-μm thick. The tissue sections were stained with hematoxylin and eosin (H&E) solution and observed with an optical microscope.

### 4.5. Statistical Analysis

All data were normally distributed and presented as the mean ± standard deviation (mean ± SD). In cases of multiple comparisons, data were analyzed using a one-way analysis of variance (one-way ANOVA) followed by Tukey’s test for multiple comparisons by Statistica software. A *p* value of less than 0.05 was considered statistically significant.

## 5. Conclusions

In conclusion, from our experimental studies, there is ample evidence supporting the fact that aluminum participates in the neuropathology of AD. The results also validate that chronic exposure to aluminum causes cognitive dysfunction and related inflammation, apoptosis and oxidative damage, which persists even after stopping AlCl_3_ administration. We evidenced that curcumin has a neuroprotective effect on animal sporadic model of AD. All our results showed a better benefit of CUR co-treatment than post-treatment, advocating its therapeutic use rather than its use in recovery.

Future research will focus on the use of the nanoparticulate/analogue approach to enhance CUR brain bioavailability in order to improve the CUR treatment strategy.

## Figures and Tables

**Table 1:** Effect of co and post-treatment by curcumin on aluminum chloride–induced a change on organ, body and food parameters in rat.

	Animal Body Weight (g)		Weight Gain (g)	Food Intake (g)	Food Efficiency Ratio (FER)	Hippocampus Weight (g)
	Initial Body	Final Body				
**CTR−**	142.45 ± 1.80	303.71 ± 2.03	62.83 ± 3.64	94.60 ± 3.42	0.19 ± 0.01	1.81 ± 0.67
**CTR+**	148.75 ± 2.96	280.75 ± 3.40 ^a^	45.08 ± 2.66 ^a^	75.37 ± 2.75	0.19 ± 0.02	1.5 ± 0.23 ^a^
**CUR1**	147.53 ± 1.67	275 ± 2.30 ^a^	36.083 ± 5.99 ^a,b^	66.43 ± 1.95 ^a,b^	0.18 ± 0.01	1.64 ± 0.26
**CTR Al**	280.75 ± 1.59	349.75 ± 2.40 ^a^	45.80 ± 2.50 ^a^	73.57 ± 2.75	0.18 ± 0.01	1.55 ± 0.19 ^a^
**CUR2**	280.50 ± 1.23	388.21 ± 1.90 ^a,^*	30.84 ± 3.14 ^a,^*	62.13 ± 1.00 ^a,^*	0.18 ± 0.01	1.63 ± 0.29

Values are mean ± SEM. *Note*: CTR−: negative control group; CTR+: positive control group (treated by aluminum chloride (AlCl_3_)); CUR1: co-treatment with curcumin; CUR2: post-treatment with curcumin; CTRAl: exposed to AlCl_3_ for 90 days and survive 60 days without any treatment; a: *p* < 0.05 as compared to CTR− group; b: *p* < 0.05 as compared to CTR+ group; *: *p* < 0.05 as compared to CTRAl group, repeated measures two-way ANOVA followed by Tukey’s test for multiple comparisons.

**Table 2:** Effect of co and post-treatments by curcumin on aluminum chloride-induced oxidative stress in rat hippocampus.

	CTR−	CTR+	CUR1	CTRAl	CUR2
**MDA**	0.62 ± 0.26	0.95 ± 0.05 ^a^	0.51 ± 0.20 ^b^	1.05 ± 0.09	0.60 ± 0.02 ^b,^*
**CAT**	0.76 ± 0.54	0.19 ± 0.03 ^a^	0.83 ± 0.26 ^b^	0.10 ± 0.09	0.89 ± 0.01 ^b,^*
**SOD**	0.10 ± 0.06	0.05 ± 0.01 ^a^	0.11 ± 0.081 ^b^	0.05 ± 0.01	0.15 ± 0.09 ^b,^*

Values are mean ± SEM. Note: CTR−: negative control group; CTR+: positive control group (treated by aluminum chloride (AlCl_3_)); CUR1: co-treatment with curcumin; CUR2: post-treatment with curcumin; CTRAl: exposed to AlCl_3_ for 90 days and survive 60 days without any treatment; a: *p* < 0.05 as compared to CTR− group; b: *p* < 0.05 as compared to CTR+ group; *: *p* < 0.05 as compared to CTRAl group, repeated measures two-way ANOVA followed by Tukey’s test for multiple comparisons.

## Data Availability

The data presented in this study are available with the authors.

## Sample Availability

Samples of the compounds are available from the authors on request.
